# Measurement of wire deflection on loading may indicate union in Ilizarov constructs, an in vitro model

**DOI:** 10.1007/s11751-018-0306-1

**Published:** 2018-02-02

**Authors:** Beth Lineham, Todd Stewart, Paul Harwood

**Affiliations:** 1 Institute of Medical and Biological Engineering, School of Mechanical Engineering, University of Leeds, Leeds, UK; 2 Department of Trauma and Orthopaedics, Leeds General Infirmary, Great George Street, Leeds, UK

**Keywords:** Ilizarov, Frame, Wire deflection, Biomechanics

## Abstract

No entirely reliable method exists for assessing union during Ilizarov treatment. Premature removal results in potential treatment failure; hence, alternative methods warrant investigation. Wire deflection might provide an indication of fracture site deformation on weight bearing, indicating progress towards union. This study aimed to test a method for assessing wire deflection within an Ilizarov frame. (1) To assess the repeatability of our novel measurement method in measuring wire deflection within an Ilizarov frame in vitro. (2) To compare the amount of wire deflection in an unstable model with that in an intact bone model. (3) To assess accuracy of this method by comparing wire deflection measured with overall machine extension. Tests were performed on clinical grade-tensioned fine wire 4-ring Ilizarov constructs stabilising a simulated fracture, with and without an unstable defect. Models were sequentially loaded to 700 N using an Instron testing machine. A digital depth gauge attached to the superior ring measured relative wire displacement at the ring closest to the fracture. Tests were repeated 3 times. (1) Both unstable and stable bone models produced highly repeatable load deformation curves (*R*^2^ = 0.98 and 0.99). (2) In the unstable model, wires tensioned at 882 and 1274 N produced mean maximum deflections of 2.41 and 2.69 mm compared with 0.05 and 0.04 mm in the intact bone model (significant *p* < 0.0001). (3) Wire deflection and machine extension results were strongly correlated (*r* = 0.99). A measurable difference in wire deflection between stable and unstable situations exists using this method which appears accurate and repeatable, with clear correlation between displacement and load and displacement and machine extension. This approach might be clinically applicable, and further clinical testing is required.

## Introduction

Ilizarov frames are increasingly utilised for the treatment of lower limb trauma and deformity. Determining bone union and the timing of frame removal can be difficult, and currently no gold standard method exists [1]. Current practice is to assess healing clinically and by serial radiographs and, sometimes, computed tomography [2]. These methods have been shown to be inaccurate and correlate poorly with fracture union and the development of complications [3]. Frames are cumbersome and difficult to live with; patients therefore prefer them removed as soon as possible. Prolonged time in a frame limits rehabilitation and can result in joint stiffness [4, 5]. Fixation elements present a potential source of infection [6], and unnecessary prolonged frame time increases complication rates [7]. Direct and indirect healthcare costs are further increased as patients are often unable to return to work with the frame in place, will attend more outpatient appointments and undergo a higher number of investigations with prolonged frame time. Thus, timely removal is desirable. However, premature frame removal before the fracture has fully consolidated can result in serious complications and treatment failure, particularly re-fracture, non-union and deformity [8]. It is desirable to investigate alternative methods to assess fracture union that could be as an adjunct to current methods.

Previous work has investigated measurement of vibration across fracture sites [9] and use of ultrasound [10] to assess union. However, ultrasound encounters problems with interference from the surrounding soft tissues and cannot provide further information on healing beyond a certain point [11, 12, 13]. Previous studies of vibration have shown errors in fractures treated with external fixation as the stiffness of the construct can be confused for stiffness of the fracture [2]. Neither of these methods is in regular clinical use currently. Mechanical assessment of fracture union has shown more promise. It is possible to measure callus stiffness across a fracture site, and such methods have shown consistent results [14, 15, 16]. Whilst these techniques are used regularly in animal studies and for pre-clinical research, such an approach has not been widely adopted clinically. Previous work has examined the potential to build stress measurement devices into the connecting rods of Ilizarov frames. In one example, an extensometer was built into distracting rods in patients undergoing femoral lengthening. The measurements obtained were used to control the elongation of the rods and assess the load distribution rather than assess union [17]. Such expensive devices would either need to be built into every frame, which would be prohibitively expensive, or be placed and removed at each clinic visit which would be time-consuming and introduces the potential to destabilise the frame during changeover. Methods which do not interfere with the fixation in any way are therefore preferable [1].

## Rationale

Weight bearing in an Ilizarov frame leads to load transfer and consequent deflection of the frame itself. This laxity is primarily due to wire deflection, since connecting rod and ring deformation has been shown to be minimal at physiological loads [18]. As the frame will act as a load sharing device with the patients’ tissues, it would seem likely that as the fracture unites, increasing fracture site rigidity will reduce movement and load transfer to the wires and therefore subsequent wire deformation [19]. If this wire deflection can be measured reliably, this variable may therefore offer a surrogate marker of bony consolidation. Whilst wire deflection has been measured using computational methods such as edge detection [20], there is, to our knowledge, no described method of wire deflection measurement that is clinically appropriate. The aim of this study was to assess the repeatability of our method for assessing wire deflection in a laboratory setting and to determine if this method could differentiate between an unstable and an intact bone reliably.

## Methods

Tests were performed on clinical grade Ilizarov equipment (Smith and Nephew). Standard 4-ring frames were constructed on a centrally mounted acrylic glass rod simulating bone. The frame was constructed using four stainless steel rings of 160 mm diameter. These rings were connected by four threaded metal rods. The simulated bone was placed slightly anteriorly in the centre of the rings to mimic the usual position achieved clinically to allow posterior soft tissue clearance. Four plain 1.8-mm Ilizarov wires per segment (8 in total) were used to mount the simulated bone with a 45°–90° crossing angle. Standard side-biting mounting bolts were used to attach these wires to the rings. Models were built either with a 4-cm bone defect to simulate a completely unstable fracture or with no defect to simulate a united fracture. Both models were constructed with wires tensioned to 882 N (90 kg) and 1274 N (130 kg). Therefore, in total four models were constructed, each model being tested 3 times.

**Fig. 1:**
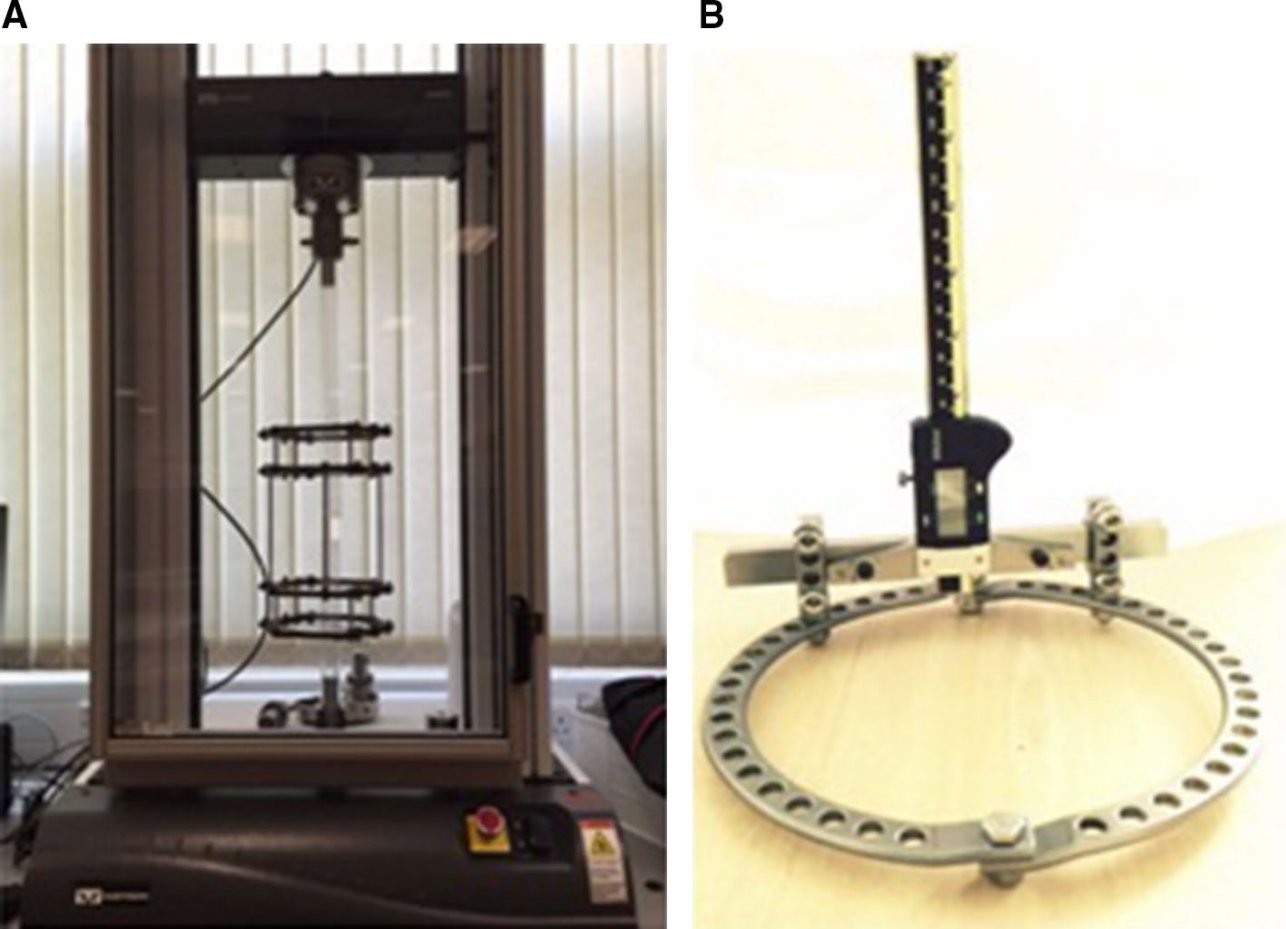
Experimental set-up showing frame for testing loaded in an Instron machine (**a**) and the depth gauge attached to an Ilizarov ring (**b**)

A depth gauge (ABSOLUTE Digimatic Depth Gage Series 571 by Mitutoyo) was attached to the most superior ring using a custom-made 316 stainless steel bracket. Measurements were taken from the most anterior wire, likely the most accessible in a clinical situation. Measurements of wire deflection were taken as close to the rod surface as possible. Models were mounted for testing in an Instron universal testing machine via the distal segment and progressive load applied in 50 N increments to the proximal segment to simulate loading the limb in the frame (Fig. 1). Wire deflection was measured at each step. Machine extension, as recorded automatically by the Instron machine, was also documented at each step. This cycle was repeated 3 times for each model. Models were preloaded prior to measurement to take account of initial wire slippage. A torque wrench was used to tension the wires.

### Statistical analysis

Measurements from each cycle of testing were taken independently for analysis. The distribution of the measured variables was examined using a Shapiro–Wilks test and found to be normal, meeting the assumptions for parametric analysis. Results are presented as a comparison of mean deflections at comparative loads on increasing wire tension. A comparison of mean maximum wire deflection between the bone defect and united model was also produced. To compare the difference between central tendency of results, the coefficient of determination was calculated using statistical software and an ANOVA test was performed. To compare the correlation of wire deflection and overall machine extension, the Pearson correlation coefficient was calculated using statistical software. A *p* value of < 0.05 was used to indicate a statistically significant difference.

## Results

**Fig. 2:**
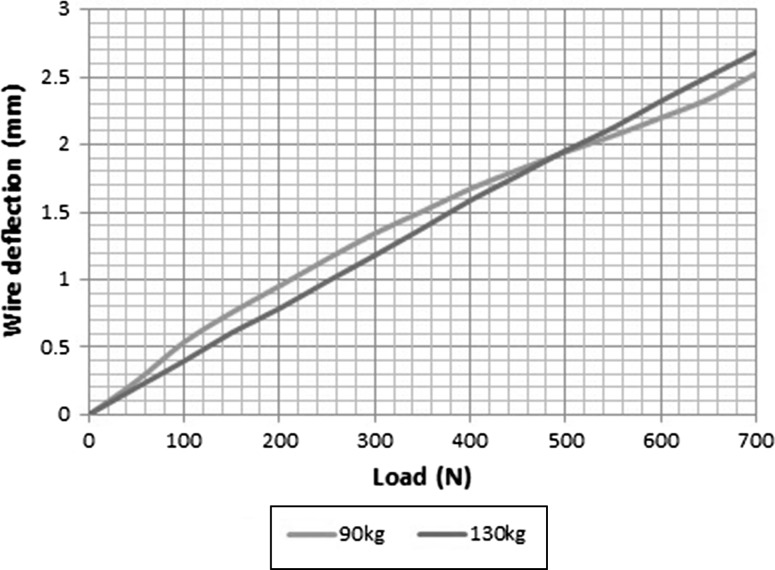
Load displacement curves for 882 N tension (grey) and 1274 N tension (black)

**Fig. 3:**
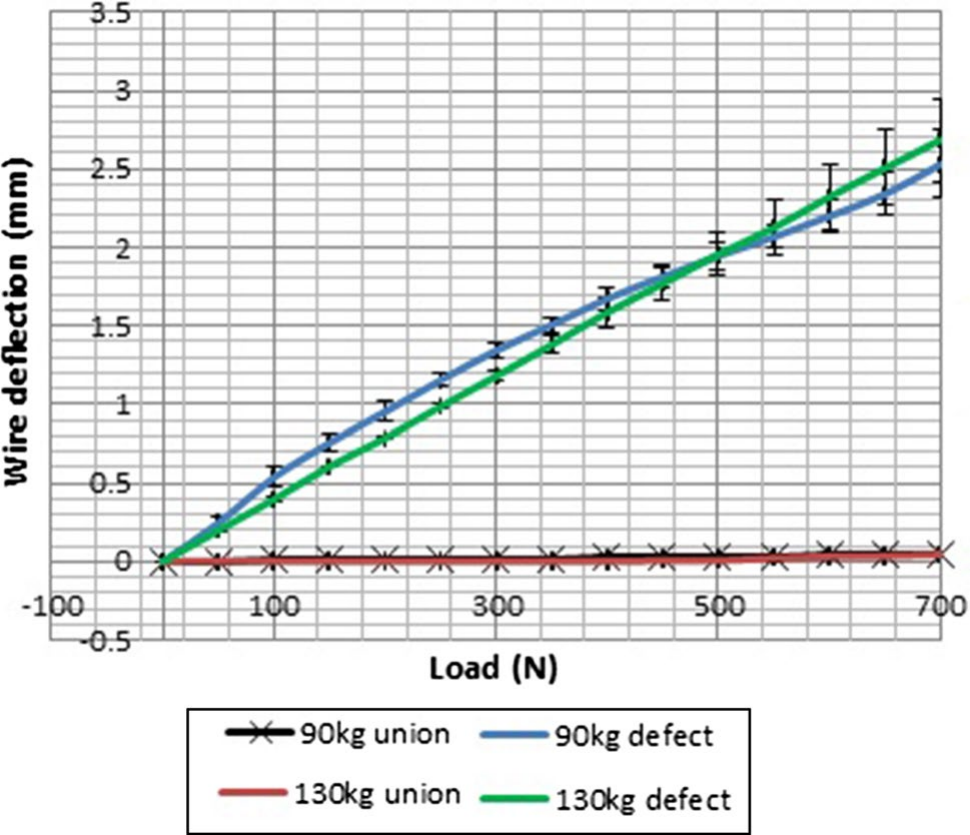
Load displacement curves for intact and unstable models at 882 and 1274 N

**Fig. 4:**
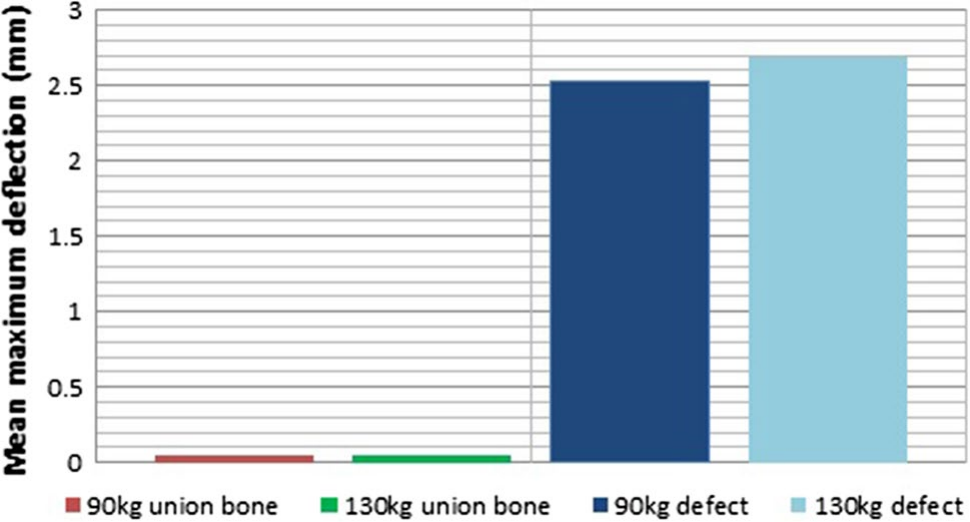
Maximum load displacement for intact and unstable models at 882 and 1274 N

Figure 2 shows load displacement curves for wire deflection at different pretensions in the unstable bone model. The mean maximum wire deflection, at 700 N load, was 2.41 mm 95% CI [2.20, 2.61] with 882 N pretension and 2.69 mm 95% CI [2.39, 2.98] with 1274 N pretension. The coefficient of determination between load and deflection was *R*^2^ = 0.98 and *R*^2^ = 0.99, respectively, for the two different levels of pretension. Testing on the simulated united bone produced much smaller deflections as shown in Figs. 3 and 4, with a mean maximal deflection of 0.05 mm at both pretensions. The difference in wire deflection between the stable and unstable models was significant (*p* < 0.0001). The strength of linear association between wire deflection and overall machine extension was significant (*r* = 0.99).

## Discussion

These results demonstrate that the described method is able to detect and quantify a difference in wire deflection between a completely stable and unstable model. The relationship between applied load and wire deflection was reproducible; this remained the case when testing wires with different pretensions. The strong correlation between wire deflection and overall machine extension indicates that the method provides a promising means of assessing fracture site motion. It is evident that this technique may have clinical application which could be used to confirm progress to bone union in patients managed with Ilizarov frames. The force applied by the patient could be measured by asking the patient to bear weight through the affected leg on a scale and the wire deflection measured using the depth gauge and comparing this with the load applied. These results would be compared over time and used to assess progress to union. In addition, complications may be identified earlier if a sudden change in the deflections are seen, indicating a decrease in overall stability.

The maximum recorded wire displacement with 884 and 1274 N of pretension was 2.76 and 2.99 mm, respectively. A previous study [20] measured the displacement of a single wire loaded perpendicular to its longitudinal axis at its mid-point, recording motion far higher than observed in this research. This is expected as the previous testing involved only one wire which was not fixed within a bone substitute. There is, to our knowledge, no previous study concerning measurement of wire deflection in a composite model. The maximum observed deflection was similar in the models tested with different levels of pretension though, interestingly, slightly higher in the 1274 N test by 0.28 mm. On examining the data further, this appeared to be due to an outlying measurement of 2.99 mm obtained in the 1274 N testing. We did not use a torque limiter to standardise bolt tightening, and as such this might have been inconsistent. We felt that this better mimicked the situation in clinical practice, but it may have led to this unexpected result if the bolts in this model were tightened less than in others. Another possibility is that at this tension plastic deformation of the wire begins to occur, increasing displacement. To investigate this, a further experiment was carried out by sequentially increasing the applied load to the wire of 1274 N pretension in 25 N increments to 800 N. No obvious change in the linear relationship between load and deformation was observed at higher loads, with the coefficient of determination reducing only slightly from 0.9995 to 0.9982. This change is so slight we cannot determine that the point of plastic deformation was reached. This agrees with results obtained in previous experimental testing. The confidence intervals obtained on repeated testing were very narrow, indicating the high precision of the measurement method used. At 884 N of pretension, the greatest margin of error was 0.19 at a deflection of 2.41 mm. At 1274 N, the greatest margin of error was 0.29 at the maximum deflection of 2.69 mm. All other measurements had margin of errors of less than 0.29 mm.

If such a device were to be used in clinical practice for assessment of fracture healing or diagnosis of non-union, it would be categorised as a medical device as per the definition from the FDA (Food and Drug Administration, 2014). As such it would have to adhere to the relevant guidelines. The MHRA (Medicines and Healthcare products Regulatory Agency, 2013) has outlined essential requirements for medical devices stating that any risks must be acceptable when compared to benefits to the patient. The risks involved with this device will be minimal; the components are non-toxic and the device does not come into contact with the patient. The device must also be compatible with substances that it may interact with. In a clinical setting, this is most likely to mean the device must be able to be cleaned using typical products, which are often an alcohol-based wipe.

## Limitations

One of the main difficulties in translating wire deflection measurement to clinical application is mounting the measurement device in the small space between the ring of the frame and the patient’s leg. Guidelines dictate that at least 2 cm of clearance should be present between the leg and each ring [21]. In testing, the clearance between the bone substitute and the ring was 4 cm and measurements were easily obtained within this space. Measurements were taken 3 mm away from the bone substitute; therefore, the device should easily fit inside 2 cm of clearance. The soft tissues will, however, mean that it is unlikely one would be able to measure deflection this close to the bone in clinical practice, which would likely reduce the measured deflection. If the device used is sensitive enough, this should, in itself, not present a problem. Additionally, if the concept is proved useful then a much smaller and specifically designed tool could be engineered for this purpose.

In this study, the same wire was measured each time, but in the different models the length of the wire from the bolt to the acrylic wire would alter the deflection measured. In clinical practice, it may not be possible to absolutely standardise the exact point of measurement on the wire.

It is important to note that wires have been shown to slip in the fixation bolts on repeated loading during treatment and lose tension over time [22]. If marked it may alter wire deflection between measurements were this method employed clinically. It will be important to take this into account during the clinical testing which has been planned.

We cannot envisage any safety risks for the examiner or the patient in undertaking of these measurements that would be above those of a normal follow-up clinic.

Data collection was carried out by one researcher and as such may be subject to observer bias depending on the reproducibility of the method. One concern in clinical application is that if the device was not placed in exactly the same manner each time the results may vary and this would need to be considered. The amount of load bearing that a patient with an Ilizarov frame can achieve also varies. This can be affected by more than just progress to union. Given the relatively linear relationship observed between load and wire deformation, it is likely that by determining the load deformation ratio (rigidity) of the wires at different time points, progressive stiffening of the system would be observable by this method despite loading of the limb changing between measurements.

A further concern over clinical application is the stability of the models used. It is difficult to model (in vitro) partially healed fractures or the contribution of soft tissues to stability accurately. The models tested were binary, being either completely unstable or completely stable, and this is not the case in clinic practice. In well-reduced fractures, there may be very little motion at the fracture site even immediately after application and therefore little change in wire deflection during progress to union. It is unclear what the effect of a uniting bone will be in different clinical situations. Certainly, this method may have application in determining union in patients with multi-fragmented fractures that have no inherent stability and in those with distraction gaps. The low-risk, non-invasive nature of the measurement technique makes such an approach attractive as a next step. Given the heterogeneous nature of the patients involved, such studies will need to be carefully designed and analysed to determine in what situations use of such a device might assist in management decisions.

## Conclusion

The purpose of this research has been to investigate a potential method to aid decision-making in determining fracture healing for patients treated with Ilizarov frames. We propose a measurement method for assessing wire displacement on loading utilising a depth gauge attached to the most proximal ring of the frame. The results obtained show a measurable difference in wire deflection between stable and unstable situations using this method, which appears valid and reliable with clear correlation between displacement and load which agrees with previous research. Clinical testing is now required to ascertain whether the displacement of the wires is equally measurable in vivo and correlates with fracture healing as we expect.
